# Identifying Sources of Lead Exposure for Children in the Republic of Georgia, with Lead Isotope Ratios

**DOI:** 10.3390/ijerph20206912

**Published:** 2023-10-12

**Authors:** Giovanni S. Leonardi, Ekaterine Ruadze, Ayoub Saei, Adam Laycock, Simon Chenery, Helen Crabbe, Elizabeth Marchant, Irma Khonelidze, Lela Sturua, Paata Imnadze, Amiran Gamkrelidze, Michael J. Watts, Tim Marczylo

**Affiliations:** 1 UK Health Security Agency, Radiation, Chemical and Environmental Hazards, Harwell Campus, Didcot OX11 0RQ, UK; adam.laycock@ukhsa.gov.uk (A.L.); helen.crabbe@ukhsa.gov.uk (H.C.); tim.marczylo@ukhsa.gov.uk (T.M.); 2 Department of Social and Environmental Research, London School of Hygiene and Tropical Medicine, London WC1E 7HT, UK; 3 Faculty of Medicine, Iv. Javakhishvili Tbilisi State University, 1 Chavchavadze Avenue, Tbilisi 0179, Georgia; e.ruadze@ncdc.ge (E.R.);; 4 The National Center for Disease Control and Public Health of Georgia, Tbilisi 0198, Georgialela.sturua@ncdc.ge (L.S.); 5 UK Health Security Agency, Statistics Unit, Department of Statistics, Modelling and Economics, London NW9 5EQ, UK; ayoub.saei@ukhsa.gov.uk; 6 British Geological Survey, Kingsley Durham Centre, Keyworth, Nottingham NG12 5GG, UKmwatts@bgs.ac.uk (M.J.W.); 7 UK Health Security Agency, Field Epidemiology Training Programme, London NW9 5EQ, UK; 8 University of Georgia (UG), 77a M. Kostava Street, Tbilisi 0171, Georgia; gamkrelidzea@gmail.com

**Keywords:** lead isotope ratio, blood lead concentration, environmental tracking, public health tracking, children, Georgia

## Abstract

In the Republic of Georgia, a 2018 national survey estimated that more than 40% of children aged 2–7 years had a blood lead concentration (BLC) of more than 5 µg/dL. The objective of this study was to document the feasibility of employing lead isotope ratios (LIRs) to identify and rank the Pb (lead) exposure sources most relevant to children across Georgia. A cross-sectional survey between November 2019 and February 2020 of 36 children previously identified as having BLCs > 5 µg/dL from seven regions of Georgia involved the collection of blood and 528 environmental samples, a questionnaire on behaviours and potential exposures. The LIRs in blood and environmental samples were analysed in individual children and across the whole group to ascertain clustering. A fitted statistical mixed-effect model to LIR data first found that the blood samples clustered with spices, tea, and paint, then, further isotopically distinct from blood were sand, dust, and soil, and lastly, milk, toys, pens, flour, and water. Analysis of the LIRs provided an indication and ranking of the importance of Pb environmental sources as explanatory factors of BLCs across the group of children. The findings support the deployment of interventions aimed at managing the priority sources of exposure in this population.

## 1. Introduction

Lead (Pb) is a widespread environmental toxicant that causes adverse health effects, such as cardiovascular, renal, and hepatic system disorders in adults [1,2,3,4,5], as well as neurological and behaviour disorders, lower IQs, slowed growth, and anaemia in children [6,7,8,9]. Lead exposure occurs in multiple settings, both indoors and outdoors, mainly through ingestion or inhalation. People can be exposed in multiple locations in their homes and/or in the outdoor environment, while for some, the workplace may present the greatest potential for exposure. Within the same location, people can be exposed via multiple sources, such as water, food, dust, or paint. Sources of Pb exposure vary across age groups. Identification of the potential major sources of Pb exposure informs the design strategy for public health interventions to completely remove or reduce the exposure or/and mitigate the Pb exposure-related health effects. The effect of public health interventions aiming to reduce lead exposure can be measured by monitoring the blood lead concentration (BLC) [10].

When used as part of an environmental health investigation, the analysis of isotopic ratios can be a tool for identifying the major source of exposure [11]. Natural Pb consists of four stable, quantifiable isotopes. The Pb isotopes of atomic masses 206, 207, and 208 are radiogenic and arise from the radioactive decay of isotopes ^238^U, ^235^U, and ^232^Th, respectively, while Pb with an atomic mass of 204 is non-radiogenic and has been present since the origin of the solar system. The abundances of Pb isotopes vary with age and original concentration of ^238^U, ^235^U, ^232^Th, and Pb of the primary Pb source [12]. During industrial processes for the preparation of Pb materials, there is no significant fractionation of isotopes, and the isotopic composition remains essentially identical to that of the original ore. Materials of different origins may have different “isotopic signatures”, i.e., different ratios between stable isotopes (lead isotopic ratios: LIRs). Recent advances in analytical techniques have increased the precision and accuracy of isotope analysis. The use of Pb isotopes has been applied to several fields, including ecology, food chemistry, forensic science, and environmental health [13,14,15,16].

In Georgia, a nationally representative sample of children between 2 and 7 years of age, selected by UNICEF in collaboration with the Georgian National Center for Disease Control (NCDC), identified a widespread exposure to Pb. The Multi-Indicator Cluster Survey (MICS) estimated that over 40% of children across the country were exposed to hazardous amounts of Pb, as represented by their BLC being greater than the current action level of 5 µg/dL [17]. In response, a national programme to reduce such exposure was established [10].

In the UK, Pb surveillance resumed in the last few years, when it became clear that, even following the removal of leaded petrol, elevated BLCs still persisted and were harmful to health. A surveillance approach based on health service laboratory data has allowed the identification of exposed children who benefit from targeted interventions [18]. In contrast, a nationally representative French sample of children was investigated with the goal of identifying several environmental Pb sources and ranking them in order of importance for the country [19]. The process of establishing such national surveillance and clarifying sources across a representative sample of children went through several phases of development and investigation [19,20,21,22,23,24,25,26,27,28,29,30,31,32,33,34,35,36]. The fact that a nationwide approach to identifying Pb sources by isotope ratio helped to address Pb exposure in children and, which was demonstrated to be feasible in France, seemed relevant to the exposure scenario in Georgia. In the absence of such an approach, any intervention aimed at reducing the Pb exposure problem would be limited by a lack of information about the relative importance of different Pb sources in driving Pb exposure in children.

The aim of this study was to use the LIR to inform intervention strategies to reduce lead exposure in children across the Republic of Georgia. To achieve this aim, our objectives were to document the feasibility of (1) investigating potential sources of lead exposure in children using the LIR and (2) identifying and ranking the lead exposure sources most relevant to Georgian children.

## 2. Methodology

### 2.1. Selection of Children

#### 2.1.1. Regions’ and Districts’ Inclusion Criteria

The data from the 1578 blood samples previously collected in the MICS was used to inform a sampling approach for this study that targeted children previously identified as having a BLC of >10 ug/dL. The MICS was conducted in 11 Georgian regions. Analysis of the BLC data of children included in the MICS showed that in five regions, Adjara, Guria, Samegrelo-Zemo Svaneti, Imereti, and Racha-Lechkhumi-Kvemo Svaneti, the geometric mean of the BLC was >5 µg/dL. In six regions, Tbilisi, Kakheti, Mtskheta-Mtianeti, Shida Kartli, Kvemo Kartli, and Samtskhe-Javakheti, the geometric mean of the BLC was <5 µg/dL. We developed slightly different sampling approaches for the regions with BLC geometric means of >5 µg/dL and <5 µg/dL. The sampling approach for the regions where the geometric mean of the BLC was >5 µg/dL was in two steps: first, within each region, the district was included if the geometric mean for the BLC was >10 µg/dL, and then within each of these districts, the child was identified as a potential participant for this study if his/her BLC in the MICS survey was >10 µg/dL—although only some of the eligible children were included in the sample due to feasibility considerations in this scoping phase of the investigation. The sampling approach for the regions where the geometric mean of the BLC was <5 µg/dL was also in two steps: first, within each region, the district was included if the geometric mean for the BLC was >5 µg/dL, and then within each of these districts, the child was identified as a potential participant for this study if his/her BLC in the MICS survey was >10 µg/dL. See included regions and districts in relation to selection criteria in Table 1. 

#### 2.1.2. Regions’ and Districts’ Exclusion Criteria

In general, if within the district where the geometric mean of the BLC was >5 µg/dL, but no child had a BLC of >10 µg/dL, the district was excluded from the study, with the exception mentioned next. Other considerations for districts’ selection were the following: (i) districts where the geometric mean of the BLC was <5 µg/dL were still included if routine data monitoring analysis showed that the Pb level in soil was above the limit value (32 mg/kg). Again, children in these districts were identified as potential participants for this study if his/her BLC in the MICS survey was >10 µg/dL, and (ii) if this condition was not met, the district was excluded from the study.

#### 2.1.3. Considerations for Child Selections within the Selected Districts

The fieldwork was coordinated with the Iashvili Children’s Hospital in Tbilisi, which participated in the State Program for MICS children with a BLC of >5 µg/dL since August 2019. If the child was a participant in this program and the final results showed that the BLC was <5 µg/dL or blood was extracted from this child within the last month before the fieldwork, this child was excluded from the study (as a blood lead of <5 µg/dL was an exclusion criterion in the proposal approved by the institutional ethical review board).

#### 2.1.4. Sample Size and Selection of Children

Due to the extensive resources required for this type of investigation of Pb sources, this study aimed to collect samples from between 30 and 40 households. As the high BLC districts (mainly from west Georgia) were of more interest, it was decided to test more households from these districts. In the districts where the BLC was not high but the Pb level in soil was found to be above the limit value, it was decided to test only one household.

### 2.2. Fieldwork

The fieldwork involved the collection of blood and environmental samples and the completion of a questionnaire. Prior signed written informed consent was obtained from each household (a parent or the guardian of the child). The team visiting the sampled households comprised a phlebotomist and a trained National Center for Disease Control (NCDC) staff member (public health specialist, epidemiologist, and/or environmental health specialist) to administer the questionnaire and collect environmental samples. The NCDC staff members were also responsible for blood transportation to the Zonal Diagnostic Laboratories and/or NCDC, as well as environmental sample transportation to the head office of the NCDC in Tbilisi. For this study’s participants, we used the same ID used in the MICS [17] to enable a time-dependent analysis of the BLC trends. If, for some reason, sample collection was not possible during the first visit, the supervisor/interviewer agreed to a suitable date for a second visit.

### 2.3. Environmental Samples Collection Approach

The soil was sampled from the gardens where children can come into direct contact with soil and also from vegetable patches/areas (if any) where vegetables/fruits are grown for home consumption. Sites for collection were obtained during the household interview and questionnaire process. Approximately 200 g of soil was collected per household and securely stored in a dry container. This soil sample comprised 10–20 subsamples randomly chosen from across the sampled area. Indoor dust samples were collected using Ghost Wipes and a 10 × 10 cm plastic template (Environmental Express, Charleston, SC, USA) following the methodology reported by Middleton et al. [37]. All wipe samples were collected from raised surfaces (e.g., window sills, shelves, tops of wardrobes). Each wipe was put in a separate, labelled tube. Paint samples were collected if there were areas of broken paint, chips, or dust from paint in the home that the child had access to. Paint samples of flakes, chips, or dust were collected in plastic, labelled ziplock bags. Unfiltered drinking water samples of 10–50 mL were collected directly into 50 mL trace-metal-grade plastic vials. If the child was known to drink water from more than one location, in addition to the tap water from the home, we took samples from other sources as well, e.g., school, nursery, relatives, etc., as it was appropriate to represent water that is drunk by the child, where they spend the majority of their time. Between 10–50 mL of milk was collected into 50 mL trace-metal-grade plastic vials and were frozen at −20 °C as soon as possible after collection. About 10–20 g of flour (maise and wheat) in use by the family at the time of the visit was collected if the child consumed products made with this flour. Samples of the flour were taken with a stainless steel spoon into plastic, labelled ziplock bags. Given the concern about spices, approximately 2–3 g of the spices used most frequently in the household, up to a maximum of 9, were collected, and further information on their use was recorded in the questionnaire. This covered a range of different individual spices and spice mixtures used extensively. We also collected different types of tea, such as tea bags, loose tea, or herbal teas.

### 2.4. Sample Transportation

All samples were sent to the UL Health Security Agency (UKHSA), except for the soil and dust and some tea samples, which were sent to the British Geological Survey (BGS) in the UK.

### 2.5. Total Pb and Pb Isotope Ratio Analysis

Total Pb and Pb isotope ratio analyses were performed by ICP-MS using the methods outlined in Laycock et al., 2022 [38].

### 2.6. Statistical Analyses

To identify the sources of exposure to Pb, the isotopic signature of the child’s blood was compared with that of the environmental samples collected in and around the child’s dwelling. The compatibility between blood and potential exposure was assessed by comparing the Pb isotope ratios (LIR) derived from the 4 Pb isotopes, taking into account the confidence intervals (CIs) formed from the LIR and measurement precision (standard error of the mean). In other words, we examined whether there was an “overlap” between the LIR CIs. A source was considered and therefore interpreted as having an explanatory value in terms of the child’s exposure to Pb from an environmental source when there was an intersection at 95% (2SE). After studying the residuals under an initial model, a log transformation was applied to the outcome data (BLC). The log-transformed data were then used as a response in the modelling.

#### 2.6.1. Discriminating Factors

In most of the studies conducted on the subject, the authors have represented the intersection based on the 2 to 3 LIRs considered the most discriminating (which makes it possible to distinguish most clearly between the sources), but without justification. In this study, we adapted the method adopted in France [19] and chose the most discriminating LIR by calculating a discriminating factor (DF) with

DF = CV/rSD

where CV is the inter-sources coefficient of variation of the LIR in the same dwelling, and rSD is the relative standard deviation (mean). The DF was calculated for each individual child. The distribution of the DF for all six LIRs was then calculated. The DF’s mean was greater than 1 for all six LIRs, as shown in Table 2. It immediately follows that each of the six LIRs can be used to investigate the association between the blood and environmental samples. However, for operational reasons, the two most discriminating LIRs were selected.

#### 2.6.2. Associations between Blood and Environmental Pb in Individual Children

To identify the major sources of exposure to Pb, the isotopic signature of the child’s blood was compared with that of the environmental samples collected, the Pb content of which is sufficient to contribute to overexposure. The compatibility between blood and the potential exposure source was assessed by comparing the LIRs, taking into account the confidence intervals (CIs) established from the LIRs and the measurement precision (2SE); whether there is “overlap” between the LIRs’ confidence intervals is examined. A source was considered compatible when there was an intersection of the LIR confidence intervals. The LIR method was found to be useful when it eliminated at least one potential source (above the threshold concentration) and sufficient when it was able to identify a single, compatible source of overexposure. In some cases, we expected to identify a relatively simple potential environmental explanation for a child’s BLC. For example, when both dust and paint from the same room were compatible with the blood, we considered that paint was the likely single source of exposure, and similarly, for the outdoor soil and dust (from the ground outside, a single source). A more complex pattern was expected for spices that were known to be often contaminated with Pb [39], although there were several types of spices of widespread use, even in this age group. To address this, an association with the group of all spices was tested, as Pb exposure could be attributed to the sum of their intake. The confidence interval calculation was based on the classical normality assumption and with independence of the variance of the mean.

#### 2.6.3. Associations between Blood and Environmental Pb in the Group of All Children

Two isotope ratios, ^208^Pb/^207^Pb and ^207^Pb/^206^Pb, with the highest DF, were selected for the group-level analysis. The design of the environmental sampling and data collection was influenced by several considerations, combining prior knowledge from the literature and preceding surveys with the local availability of samples. Overall, this process could be described as an opportunistic sampling of several potential environmental sources of Pb exposure rather than a systematic collection of the same environmental samples for each child. Given this structure of the data, a two-stage statistical method was used to rank the Pb sources across the group. The data underlying the modelling were the LIR means. A log transformation was applied to the standardised LIR means first; then, the two LIRs were jointly modelled in a single model. We used a shared random effect approach to induce a correlation between the two responses. The model included the region and lab in addition to the environmental sources’ random effect. The environmental-source random effects were allowed to be correlated. The first step used a generalised linear mixed model (SAS GLIMMIX procedure), as this was expected to fit the statistical models to data with correlations or nonconstant variability and where the response was not necessarily normally distributed. These models are known as generalised linear mixed models (GLMM). The second step was to use the predicted values of the environment samples to ascertain the clustering of Pb environmental sources with the BLC values to provide an indication and ranking of the importance of the Pb environmental sources as explanatory factors of BLCs in children across the group of children.

The final fitted model was:

$$\mathrm{Model}\text{:}\;y_{c(i)t}={x^{\prime }}_{c(i)t}\beta +u_{it}+e_{cit}$$

where $x_{c(i)t}$ is a vector of *p’s* known regression variables with the regression coefficient **β,** and $u_{\mathrm{it}}$ is the bivariate normal with a zero mean, variances of $\varphi_{c1},\varphi_{c2}$, and the covariance of $\varphi_{c3}$ LIR *t* (1 = ^208^Pb/^207^Pb and 2 = ^207^Pb/^206^Pb), *i* = 1,2, …, 12 and *c* = 1,2, …, 36. The $e_{cit}$ are the model errors with zero means and variances of $\sigma_{1}^{2}$ and $\sigma_{2}^{2}$, respectively.

Regression-type models are the most often applied approaches for studying an association between blood and environmental-source LIRs. However, the particular data collection design with environmental sources differing between children made the association study challenging; therefore, the environmental sources were regrouped into a small group of four sources: dust/paint (x1), soil/sand (x2), spices (x3), and food/toy/pen/other (x4). The outcome/response of interest was the isotope ratio mean. Data were also available on the standard deviation and, as a result, the standard error of the LIR mean. The relationship between the blood LIR and the four environmental sources was examined by scatter graphs for ^208^Pb/^207^Pb and ^207^Pb/^206^Pb, respectively (not shown), indicating a positive but not-so-strong association between the blood and environmental sources. A series of regression-type models were developed to estimate and test this association. Initial models 1, 2, and 3 were based on the assumption of homoscedasticity of the residuals and other regression assumptions. Model 4 relaxed the independence assumption between the variance and mean, and the centring and scaling helped fit this model and allowed for a correlation between the centred and scaled dependent variables (LIRs ^208^Pb/^207^Pb and ^207^Pb/^206^Pb) by including a shared child random effect. The residual variance was modelled as a function of the mean. A series of models were developed by using further information on the standard deviation (and, as a result, on the standard error) of the LIR mean of runs rather than a single observation. Model 5 used weights based on the inverse of the variance of the blood LIR mean estimator, and the observations used in this model were weighted appropriately according to their variation. Model 6 extended Model 5 by allowing a dependence of the residuals’ variance on the mean and also relaxed the independence assumption in Model 5 by introducing a shared child random effect into the model.

## 3. Results

### 3.1. General Description of the Children Sample

**Figure 1:**
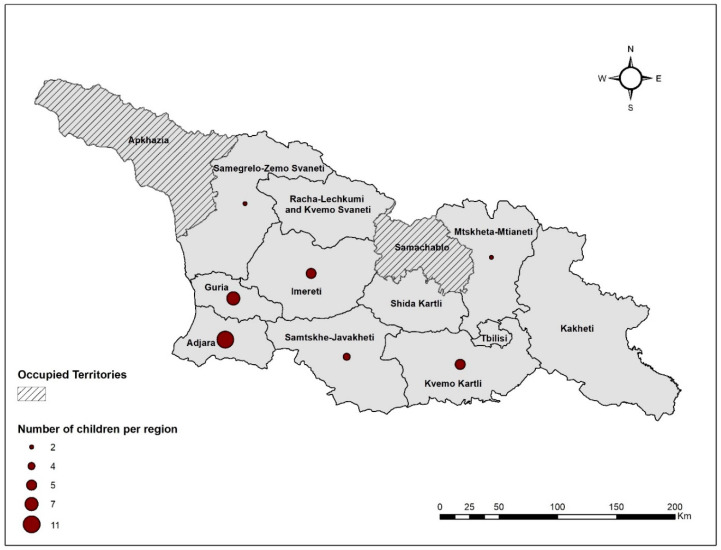
Geographical distribution of children sampled in Georgia (*N* = 36).

The field visits took place from November 2019 until February 2020. Data were collected in two rounds. During the first visits, November–December 2019, the questionnaire was completed, and all samples were collected except for the dust and soil samples. During the second round of fieldwork, January–February 2020, dust and soil samples were collected. The final sample set consisted of 36 children from seven regions of Georgia (Figure 1).

The minimum age of the children at the interview was 3 years, and the maximum was 11 years. The median age was 7 years (IQR 4 years–8 years). Of the 36 children, 19 (53%) were male.

### 3.2. Blood and Environmental Samples

In total, we collected 528 samples of 14 different types. Table 3 provides the distribution of the environmental samples collected in the study.

Blood samples were taken from all 36 children included in the study. The minimum BLC was 2.6 µg/dL, and the maximum was 39.9 µg/dL. The median BLC was 12.5 (IQR 8.3 µg/dL–18.9 µg/dL). Only two of the selected children had a BLC below the reference value (5 µg/dL) but were chosen because of the soil’s Pb concentration. In 12 (33%) children, the BLC was between 5 µg/dL and 10 µg/dL. In 22 (61%) children, the BLC was above 10 µg/dL.

Although the BLC remained high, a decrease in BLC was observed in 85% (*n* = 29) of the children when compared to the MICS data of 2018. The maximum decrease was 15 µg/dL, and the minimum was 0.9 µg/dL. The median decrease was 5.8 µg/dL (IQR 8.6 µg/dL–4.1 µg/dL).

In one household with two children, the guardian consented only to take the blood from the sibling and not from the child included in the MICS.

### 3.3. Descriptive Analysis of the Environmental Samples

Of the sampled spices, Pb levels above the reference value (5 mg/kg) were mainly found in blue fenugreek (11 samples out of 14, 78.6%), yellow flower (7 samples out of 13, 53.8%), and in spice mixes (13 out of 20, 65.0%).

Thirty-two households out of the thirty-six (88.9%) mentioned that the child eats the same food as the parents, including food cooked with spices.

Lead levels above the limit value were found in 54% of paint, 43% of spices, 25% of soil, and 10% of dust samples (Table 4).

### 3.4. Lead Isotope Ratio Analysis of Individual Cases

The LIR analysis at the individual child level showed that the Pb ratios from the spices contributed to the ratios of the blood in 72% (26/36) of children, dust Pb isotope ratios contributed in 53% (19/36) of children, paint in 11% (4/36) of children, toys in 11% (4/36) of children and tea in 3% (1/36) of children. In 14% of children (5/36), we did not find a clear close association between the isotope ratios from the environmental samples and those from the blood. The isotope ratio findings for each of the children are provided in Table 5.

### 3.5. Lead Isotope Ratio Analysis of the Group of Children

**Figure 2:**
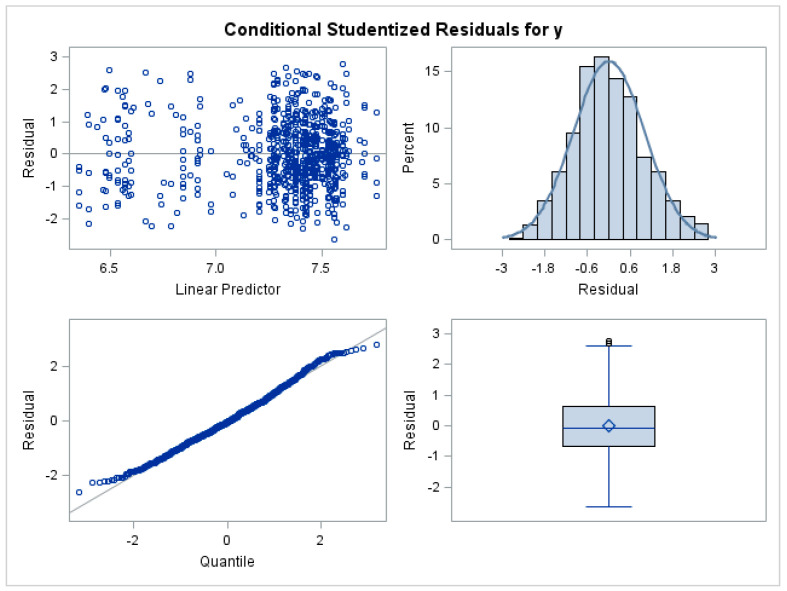
Model diagnostic plots of the final model fitted.

Analysis of the residuals for the generalised linear mixed model showed that this is an acceptable approach for the analysis of these data with correlations and nonconstant variability and that the use of log-transformed means is required; see Figure 2. The plots in Figure 2 are model diagnostic plots of the final model with two correlated random effects and adjustments for the region and lab. The diagnostic plots demonstrate that the results did not show any serious violation of the model assumptions.

**Figure 3:**
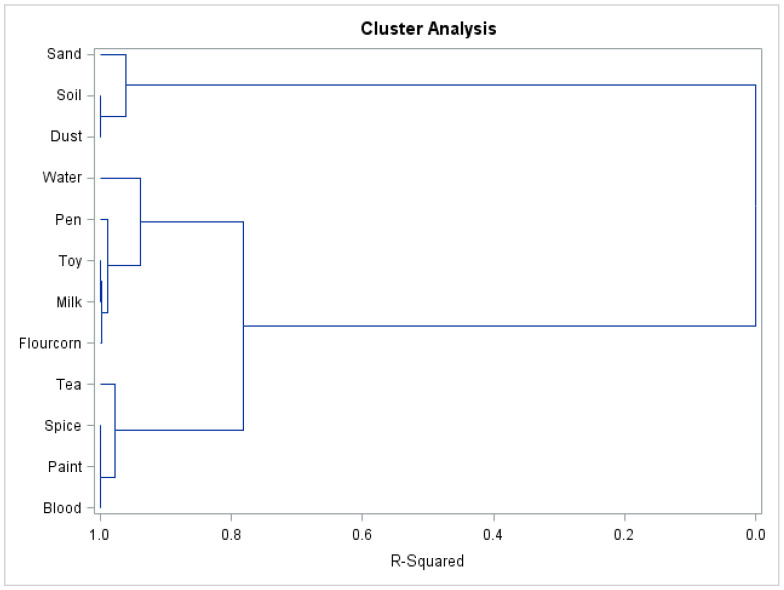
Cluster analysis of Pb isotope ratio in blood and environmental samples in children of Georgia.

The results of the clustering of Pb environmental sources with the BLL values under the final model are summarised in the following plot (Figure 3).

We considered several possible numbers of clustering of Pb environmental sources. When two clusters are considered informative, dust, soil, and sand are grouped in cluster 1, and all other environment samples, such as milk, toys, paint, spice, flour, pen, tea, and water, are grouped with blood in cluster 2. When three clusters are considered, milk, toy, pen, flour and water are in cluster 1, sand, dust, and soil are in cluster 2, and spice, tea, paint, and blood are in cluster 3. Under four-cluster clustering, water itself is a cluster (cluster 4), paint, spice, tea, and blood are in cluster 3, dust, soil, and sand are in cluster 2, and milk, toy, flour, and pen are in cluster 1. For five clusters, it is interesting to note that sand leaves cluster 2 under four-cluster clustering and makes a new cluster under five-cluster clustering. The other clusters do not change between the four and five-cluster clustering. The same general conclusion, in terms of model fits and clustering the environmental sources with the BLL values, would be obtained if we had used LIRs ^207^Pb/^206^Pb and ^206^Pb/^204^Pb rather than LIRs ^208^Pb/^207^Pb and ^207^Pb/^206^Pb.

The results of clustering of Pb environmental sources with BLC values provide an indication that the Pb sources with higher explanatory power are spices (grouped with the inclusion of tea), followed by dust and paint sources (grouped with water and milk sources), whereas the explanatory power of the sand and soil sources is minimal in this group; see Figure 3.

The regression model results show significant variation between children and a dependence of residual variance on mean, consistent with a Pb exposure pattern reflecting a mixture of chronic baseline exposure and acute anomalous exposure across the group. Table 6 presents the fit results for Models 5 and 6. The conclusions about the association between the blood and environmental sources differed from those of Models 1, 2, 3, and 4, showing strong evidence for a significant association. In addition, Models 5 and 6 included information on the standard deviation of the LIR sample runs, and Model 6 further assumed that the influence of Pb exposure sources is different between children. Based on Model 6, the estimates of the influence of Pb exposure groupings rank spices as the most influential, followed by dust and paint exposure sources, and then other foods and objects available to the child. The negative coefficient for the soil and sand exposure sources is estimated within a confidence interval crossing zero and indicates no obvious influence of this last group of Pb exposure sources on the exposure of children, as represented by their blood LIRs. Note that the regression model only used data for 24 children, and regrouping of the environment samples was unable to include data for all 36 children in the model. In addition to that, the sampling was neither random nor representative.

## 4. Discussion

Several potential Pb sources, such as drinking water, milk, flour, and contaminated soil, were ranked low as an explanation of elevated children’s BLCs, whereas others, such as dust, paint, and spices, were ranked high overall. This result provides an insight into the practical relevance when developing strategies for the management of Pb exposure across this population, as it allows health agencies to prioritise interventions directed at highly ranked Pb environmental sources most likely to explain children’s exposure across the country. Pb isotope ratio analyses are sometimes perceived as being complex to conduct and interpret and, therefore, used as a supplementary tool in the evaluation of individual household lead hazards but are not often applied to public health analyses [13,40]. The fact that potential lead sources (e.g., paint, soil, dust) plot in linear arrangements with the biological (e.g., blood) samples does not necessarily allow the distinct identification of the actual lead sources to the child., as there is no certainty that all actual sources have been sampled and because there are multiple possible combinations of these sources that could produce the isotopic value of the biological sample [41]. However, the fact that lead isotopes are to be measured does not affect the sampling procedures or the number of samples taken. The same authors recommend that a thorough evaluation of household lead hazards would benefit by incorporating (a) the lead concentrations and loadings in the household environment, (b) all isotopic ratios of potential lead sources, and (c) information about behavioural habits, as well as an evaluation of the viable pathways of exposure to the child [13]. These recommendations have been applied successfully in several contexts, such as in children of rural Bangladesh [41], in three regions of Japan [42], and in Western USA [43].

Our study documents the feasibility of implementing a Pb isotope ratio analysis to a population sample of children exposed to Pb from multiple sources of unknown relative importance, provided this work is designed and implemented by a multi-disciplinary team with experience in public health systems, environmental epidemiology, environmental health fieldwork, analytical chemistry, and toxicology applied to health protection. Therefore, it has been feasible to apply Pb isotope ratio analyses alongside the total Pb analysis for the ranking of multiple environmental sources of Pb exposure in terms of their explanatory power of BLCs in a group of children in Georgia. In a group of children found to have been exposed to Pb and with relatively elevated Pb blood concentrations, the isotope ratio analysis indicates which Pb exposure sources in the environment have had an important or negligible influence on the Pb exposure of that group. The results contribute to a new direction in the application of lead-stable isotope ratio measurements for identifying and then reducing prevalent lead sources associated with high BLCs [16].

Analyses based on grouping Pb exposures by the “logical components” of the overall Pb exposure had several limitations. The types of samples collected differed across households, and information was lost when we collapsed all LIRs into groups. There may be several reasons for this; the main one may be that the LIR information is specific to individual samples, and the computed means are based on a loss of information provided by the individual environmental samples.

There were 32 types of environmental samples tested for the LIR across this study, and the number and type of environmental samples differed between children. This is a limitation of the study, as the information obtained in the group-level analysis could not be referred directly to the whole group of children and, therefore, has a mainly exploratory value. The regression conclusion can be tested in a larger sample survey of Georgian children. The results can be expanded to all children in Georgia by using data from a large and representative sample survey of children.

If a specific environmental source was demonstrated to have a large explanatory power in terms of clustering with the BLC, this does not mean that that source is generally important to address across the whole population, as only children with relatively high BLCs were investigated here. The public health implications of these findings for this group of children were to increase confidence to direct specific interventions to reduce exposure to Pb. At the household level, interventions, including communication of the results and health promotion, were provided by and focused on reducing exposure to specific sources relevant to the individual household. Both written communication and a visit to the household were provided by the public health service in collaboration with the authors. At the level of the group as a whole, the results informed the debate about the ranking of importance of Pb exposure sources in children across the regions of Georgia. To confirm its relevance to national public health policy, this methodology should now be applied to a sample representative of the whole population of children in Georgia.

The results based on grouping Pb sources provide a meaningful simplification of the actual complexity of Pb exposures. Also, the same five groups were based on topics that were relevant as explanatory terms for the presentation of individual sources. Isotope ratio analysis at the group level was useful for ranking the importance of Pb environmental sources as explanatory factors of BLCs in children across this group of children. The interventions required to reduce Pb exposure vary with each type of Pb source. For example, interventions appropriate to reduce exposure to Pb in spices would focus on further elements to clarify the mechanisms by which spices are contaminated and how such contamination could be removed or reduced, whereas interventions to reduce exposure via paint and dust would clarify the implementation of existing regulations on Pb in paint and perhaps further exploration of the sources driving dust Pb concentrations. Therefore, the key value of the isotope ratio analysis demonstrated in this study was to rank and prioritise the Pb sources most relevant for future targeted interventions. Regarding exposure to Pb via spices, this is a complex topic of potential relevance beyond Georgia; see, for example, the identification of turmeric as a source of Pb exposure in children in the USA [43]. For population studies of this topic, knowledge of the drivers of regional variations in consumption of individual spices as well as their Pb content and isotopic composition to confirm provenance, will also be valuable.

### 4.1. Next Steps

In the context of a population with widespread exposure of children to harmful concentrations of Pb, it would be appropriate to assess the environmental Pb sources as explanatory factors for BLCs within a representative sample of all children potentially exposed. The complexity of such an activity is related to the need to define the criteria for the selection of children, which produces representativeness, and then conduct several data collections and analyses focused on Pb concentrations in several environmental media as well as blood. The inclusion of Pb isotope ratio analyses is key to producing evidence on the relative importance of diverse environmental sources, thus representing a step toward prevention activities targeting the whole exposed population and, thus, not confined to individual cases. Identifying exposed children from a proactive national surveillance programme could produce observations valid across the entire population, including for age and area-specific strata, whereas if drawn from specific clinics or locations, the observations would be relevant only to the population served by such clinics or of those locations. Also, observations from a passive surveillance system, designed to capture events as reported by clinicians aware of Pb exposure, could not represent exposure across the population.

### 4.2. Conclusions

The LIR is a valid method for identifying sources of lead in children that could be used in Georgia and allows for the prioritisation of sources in order of importance for human health. Preventative actions must target household settled dust and dietary spices and further explore the role of old paint, tea, and other food items to reduce the BLCs of children in Georgia.

The relative importance of specific exposure routes across a nationally representative sample of children could be ascertained to ensure the deployment of interventions aimed at managing the priority sources of exposure in this population.

## Figures and Tables

**Table 1:** Initial selected regions and districts based on the selection criteria.

Region	District	Mid-Year Population 2021 ^1^	BLC Arithmetic Mean (µg/dL)	BLC Geometric Mean (µg/dL)
Adjara	Khulo	26,700	17.5	15.4
Adjara	Khelvachauri	52,700	13.6	11.9
Adjara	Kobuleti	71,300	12.2	12.1
Adjara	Shuakhevi	14,900	17.3	15.0
Guria	Chokhatauri	17,700	12.3	9.6
Guria	Lanchkhuti	29,700	11.2	9.4
Imereti	Zestaponi	54,700	10.7	9.1
Imereti	Chiatura	37,900	14.5	10.8
Imereti	Tkibuli	17,500	11.5	7.7
Samegrelo	Abasha	19,300	15.1	12.1
Samegrelo	Chkhorotsku	21,200	10.3	9.4
Samtskhe	Adigeni	16,000	5.7	5.0
Samtskhe	Akhaltsikhe	39,300	5.2	4.2
Samtskhe	Aspindza	10,600	8.0	5.7
Kvemo Kartli	Tsalka	19,700	16.8	16.8
Mtskheta-Mtianeti	Kazbegi	3800	5.8	5.5
Mtskheta-Mtianeti	Tianeti	10,200	9.3	6.3
**Regions with high soil concentrations**				
Kvemo Kartli	Gardabani	79,800		
Kvemo Kartli	Marneuli	107,700		
Kvemo Kartli	Bolnisi	56,000		No child > 10 µg/dL
Kvemo Kartli	Dmanisi	20,900		No child > 10 µg/dL
Kakheti	Dedoplistskaro	20,500		No child > 10 µg/dL
Imereti	Sachkhere	34,500		

^1^ Mid-year population by regions and self-governed units: https://www.geostat.ge/en/modules/categories/41/population; accessed on 6 July 2022.

**Table 2:** Discriminating factors for LIR analyses.

Discriminating Factor (Related to Pb Isotope Ratio)	Obs.	Mean	SD	Min	Max
DF1 (^208^Pb/^207^Pb)	36	3.5	1.8	1.3	10.7
DF2 (^208^Pb/^206^Pb)	36	2.7	1.0	1.2	5.8
DF3 (^208^Pb/^204^Pb)	36	2.8	1.6	1.0	6.9
DF4 (^207^Pb/^206^Pb)	36	6.1	2.2	2.9	12.4
DF5 (^207^Pb/^204^Pb)	36	2.0	1.9	0.5	10.2
DF6 (^206^Pb/^204^Pb)	36	4.4	2.1	2.2	13.2

**Table 3:** Types and number of environmental samples (*n* = 528) collected for the identification of Pb exposure sources.

	Sample Type	Detailed List	No of Samples
1	Blood		36
2	Wheat or corn flour		62
		Wheat flour	36
		Corn flour and corn	26
3	Kinetic sand		1
4	Milk	Milk, home	15
5	PLAY-DOH		1
6	Paint		22
		Paint, indoor	19
		Paint, outdoor	3
7	Pen	Plastic pen	6
8	Sand		2
		Sand from playground	1
		Sand from yard	1
9	Soil		60
		Soil from vegetable garden	31
		Soil from the yard	26
		Soil from school	1
		Soil from other homes	1
10	Spice		136
		Red pepper (dried)	13
		Coriander (seeds)	16
		Blue fenugreek	14
		Saffron/yellow flower	13
		Black pepper (packed)	13
		Seasoned salt (svanuiri salt)	11
		Rosemary	10
		Cumin	6
		Adjika/homemade	8
		Other individuals	6
		Mixes	20
		Red pepper (whole vegetable)	6
11	Tea		31
12	Toy		16
		Not-paint covered toy	12
		Paint covered toy	4
13	Water		48
		Water mains (residential)	17
		Water private (well, spring) (residential)	23
		Water, school	7
		Water, other	1
14	Dust		92
	Total		528

**Table 4:** Pb concentration in blood and environmental samples.

	Sample Type	Unit of Measurement	Reference Value	# of Samples	No of Samples Exceeded Reference Value (*n* (%))	Range (min–max)	Median [25–75% IQR] of Pb
1	Blood	µg/dL	5	36	34 (94.4)	2.6–39.9	12.5 (8.3–18.9)
2	Spices	mg/kg	5	136	59 (43.4)	0.01–6165	2.88 (0.28–22.6)
3	Wheat and corn	mg/kg	0.5	62	1 (1.6)	0.003–1.49	0.008 (0.006–0.015)
4	Kinetic sand	µg/kg	2000	1	0 (0.0)	28	N/A
5	Milk	µg/L	20	15	1 (6.7)	7.1	N/A ^1^
6	PLAY-DOH	µg/kg	2000	1	0 (0.0)	21.7	N/A
7	Indoor paint	mg/kg	90	19	11 (57.9)	0.8–4801	32.9 [(3.1–367.6)
8	Outdoor paint	mg/kg	90	3	1 (33.3)	2.9–161.2	4.9 [2.9–161.2]
9	Pen	µg/kg	23,000	6	0 (0.0)	8.4–652	344.2 [178.3–632.2]
10	Sand	mg/kg	32	2	0 (0.0)	10.9–12.8	N/A
11	Soil	mg/kg	32	60	15 (25.0)	9.2–158.8	23.7 (17.9 36.6)
12	Tea	mg/kg	10	31	0 (0.0)	0.07–2.1	0.4 (0.29–0.59)
13	Toy	µg/kg	23,000	16	0 (0.0)	1.8–91.6	33.1 (13.3–80.1)
14	Water	µg/L	10	48 ^2^	0 (0.0)	0.02–0.29	0.06 (0.03–0.13)
15	Dust	µg/ft2	40	92	9 (9.8)	0.4–201	8.2 (3.2–15.7)

^1^ In 14 milk samples, the lead level was below the limit of detection. ^2^ In 29 water samples, the lead level was below the limit of detection.

**Table 5:** Summary of association between blood Pb and environmental sample Pb based on LIR compatibility.

			Number of Samples					
Child ID	Region	BLC/µg/dL	Dust (D) and/or Paint (P)	Soil and/or Sand	Spices	Other Foods	Other	Closest Association in Terms of LIR
1	Mtskheta-Mtinaneti	8.7	3 (D)	2	7	6	0	Dust
2	Mtskheta-Mtinaneti	14.0	4 (D/P)	3 (SS)	7	6	0	Spices
3	Kvemo Kartli	14.5	1 (P)	2	7	4	0	Spices
4	Kvemo Kartli	14.8	4 (D)	1	0	3	0	Dust
5	Kvemo Kartli	2.6	3 (D)	1	4	4	0	Dust and spices
6	Kvemo Kartli	4.2	3 (D)	1	0	4	0	Dust
7	Kvemo Kartli	6.6	5(D/P)	1	7	4	0	Dust
8	Samtskhe-Javakheti	8.0	4 (D/P)	1	5	5	0	Tea and dust
9	Samtskhe-Javakheti	9.8	5(D/P)	1	5	5	0	Spices and dust
10	Samtskhe-Javakheti	6.5	4 (D/P)	2	8	6	0	Spices and paint;
11	Samtskhe-Javakheti	5.0	5(D/P)	2	6	7	0	Spices
12	Adjara	6.2	3 (D)	2(SS)	2	3	0	Spices
13	Adjara	39.9	4 (D/P)	1	4	3	0	Not found
14	Adjara	18.1	3 (D)	2	5	2	0	Dust
15	Adjara	18.6	3 (D)	2	6	6	0	Dust and spices
16	Adjara	10.9	3 (D)	2	2	6	0	Dust
17	Adjara	12.7	0	1	6	2	0	Spices
18	Adjara	12.6	0	2	1	4	0	Not found
19	Adjara	10.8	2 (P)	2	13	4	0	Spices
20	Adjara	8.7	4 (D/P)	2	5	5	1	Dust and spices
21	Adjara	12.5	3 (D)	2	0	4	2	Dust
22	Adjara	8.8	3 (D)	0	3	5	2	Not found
23	Guria	26.3	3 (D)	3	0	3	5	Soil samples and Toys
24	Guria	11.3	5(D/P)	1	5	3	1	Spices, dust samples, and toys
25	Guria	9.4	4 (D/P)	2	4	4	0	Paint
26	Guria	27.4	4 (D/P)	2	4	4	2	Dust samples and toys
27	Guria	25.0	4 (D/P)	2	3	4	2	Spices
28	Guria	23.0	4 (D/P)	2	2	4	4	Spices, dust, and paint and toy samples
29	Guria	13.1	3 (D)	2	3	6	1	Dust and spices
30	Samegrelo	19.2	1 (P)	2	3	4	0	Paint
31	Samegrelo	7.6	1 (P)	1	1	5	3	Spices
32	Imereti	6.7	3 (D)	2	0	4	0	Not found
33	Imereti	30.1	3 (D)	2	2	5	1	Not found
34	Imereti	14.6	2 (D)	2	1	3	0	Spices and dust
35	Imereti	25.2	4 (D/P)	2	2	4	0	Dust
36	Imereti	28.8	4 (D)	2	3	5	0	Spices

**Table 6:** Regression results on the role of LIR for Pb exposure sources in 4 groups on the blood LIR.

	Model 5				Model 6			
Parameter	Est	P-V	LC	UC	Est	P-V	LC	UC
Intercept 1	1.60	0.00	0.77	2.44	0.56	0.00	0.42	0.69
Intercept 4	0.75	0.00	0.51	0.98	0.69	0.00	0.48	0.90
Dust/paint 1	0.24	0.06	0.00	0.49	0.24	0.00	0.16	0.31
Dust/paint 4	−0.03	0.73	−0.22	0.15	0.05	0.52	−0.10	0.20
Soil/Sand 1	−0.14	0.12	−0.31	0.03	−0.15	0.00	−0.22	−0.07
Soil/Sand 4	0.05	0.41	−0.06	0.16	0.04	0.28	−0.03	0.11
Spices 1	0.17	0.16	−0.06	0.41	0.50	0	0.45	0.56
Spices 4	0.05	0.54	−0.10	0.19	0.04	0.65	−0.15	0.24
Food/toys/pen/other 1	0.06	0.56	−0.15	0.27	0.18	0.00	0.14	0.21
Food/toys/pen/other 4	0.08	0.39	−0.10	0.26	0.07	0.39	−0.09	0.22

Est = Estimate, P-V = p-value, LC = lower limit and UC = upper limit of 95% confidence interval.

## Data Availability

Data underlying the findings described in the manuscript will not be shared in view of personally identifiable information required for analysis.
